# Immunogenicity and Protective Efficacy of an mRNA Vaccine Targeting HSV-2 UL41 in Mice

**DOI:** 10.3390/vaccines13030271

**Published:** 2025-03-05

**Authors:** Tangwei Mou, Yu Zhao, Jie Jia, Kai-Cheng Gao, Shao-You Li, Yi-Qun Kuang

**Affiliations:** Research Center for Clinical Medicine, First Affiliated Hospital of Kunming Medical University, Kunming 650032, Chinazhaoyu_guapier@163.com (Y.Z.);

**Keywords:** mRNA vaccines, HSV-2, UL41 protein, immune response, protective effects

## Abstract

Background: Herpes simplex virus 2 (HSV-2) is the primary cause of sexually transmitted genital ulcerative diseases, for which no effective prophylactic vaccine is currently available. However, the identification of appropriate targets for an HSV-2 mRNA vaccine remains an area requiring further investigation. Methods: The immunogenicity and protective effects of an HSV-2 UL41 mRNA vaccine were evaluated in a BALB/c mouse model. The mice were intramuscularly immunized twice, followed by HSV-2 infection at 28 days post boost. Clinical signs were monitored daily, and the viral load and tissue inflammation were assessed on days 1, 4, and 7 post infection. Dendritic cell (DC) activation in spleen tissue was analyzed via transcriptome sequencing. Results: A comparison of the clinical, immunological, and pathological characteristics of the groups that were immunized with the UL41 mRNA vaccine and then infected with HSV2, along with the control groups, revealed that the vaccine elicited both cellular and humoral immunity, inhibited viral replication, suppressed the inflammatory response, and provided protective effects against the virus in vivo. Furthermore, in vitro assays of DC expansion revealed that the vaccine immunization increased the induction of DCs from splenic cells. Transcriptomic analysis of these DCs revealed the activation of immune signaling pathways. Conclusions: Our study suggests that the UL41 mRNA vaccine may provide effective protection against HSV-2-related diseases and holds promise as a potential mRNA vaccine candidate.

## 1. Introduction

Herpes simplex virus type 2 (HSV-2) infection is a highly prevalent infectious disease which is the primary cause of the majority of clinical cases of genital ulcers worldwide and significantly impacts neonatal mortality [1,2,3,4]. Following infection, HSV-2 can be reactivated repeatedly, with approximately 60% of patients experiencing recurrent episodes [5]. Notably, 20% of individuals may suffer from more than 10 recurrent genital ulcers within the first year post infection [6]. Additionally, vertical transmission of HSV-2 can occur during pregnancy [7,8]. Furthermore, HSV-2 infection increases the global incidence of HIV infection three- to six-fold [9,10]. Consequently, the periodic recurrence of HSV-2 complicates disease management and prolongs recovery, severely affecting the physical and mental health of patients [2,11]. Thus, early prevention and treatment of genital herpes are clinically important for controlling the spread of HSV-2 and mitigating its detrimental effects.

Currently, the vaccines that have been extensively studied and clinically evaluated include live attenuated vaccines, replication-defective virus vaccines, DNA vaccines, subunit vaccines, and peptide vaccines [12]. However, these candidates remain largely in the early research or clinical trial stages. While significant progress has been made in animal models, each vaccine type presents its own set of advantages and disadvantages [13,14,15,16,17,18,19,20]. There is currently no ideal HSV-2 vaccine available on the market.

The natural immune system is the first line of defense against pathogenic infection in the host, and it is also the sentinel for specific immune responses to detect pathogen invasion [21]. The host immune system plays a crucial role in preventing and controlling HSV-2 infection, as well as minimizing the severity of the diseases it causes. Both innate and adaptive immunity are involved in protecting the host from HSV-2 [22]. These mechanisms involve a variety of viral proteins, especially immediate–early proteins, envelope proteins, and other proteins that target cellular antiviral signaling pathways [23]. In the development of HSV-2 vaccines, glycoproteins gD, gB, gH, gL [13], tegument protein VP16 [24], and capsid protein VP5 [25] are extensively studied as key targets. These proteins play critical roles in the viral life cycle and significantly influence immune system recognition and neutralization, making them central to various vaccine strategies. However, the potential effects of HSV-2 capsid proteins as vaccine targets remain unclear. Capsid proteins primarily function in forming the virus’s protective shell of viruses and are essential for their structural integrity. Unlike viral glycoproteins, which are directly involved in virus–host cell interactions, capsid proteins do not engage in this process. While capsid proteins exhibit some degree of immunogenicity, their internal location within the virus may limit their recognition by the immune system through conventional immune responses. Despite these limitations, capsid proteins may have potential roles in inducing robust cellular immune responses, enhancing viral clearance, and providing long-term vaccine protection.

The capsid protein encoded by UL41 is an endoribonuclease, also known as virion host shutoff protein (VHS), which can degrade host mRNAs, such as cGAS mRNA, to prevent the cGAS/STING-mediated DNA recognition pathway [26]; it can also target the zinc finger antiviral protein (ZAP) to degrade it, a host cell restriction factor that has antiviral activity, and inhibit its expression to achieve immune evasion [27].

To further explore the role of the UL41 mRNA vaccine in protection against HSV-2 infection, this study will clarify the immunogenicity of the HSV-2 UL41 mRNA vaccine, evaluate the effect of the HSV-2 UL41 mRNA vaccine in preventing diseases associated with HSV-2 infection, and elucidate the roles of the UL41 mRNA vaccine in the immunological mechanism against HSV-2-related diseases. Our exploration provides a new theoretical basis for the prevention and treatment of recurrent herpes caused by HSV-2 infection.

## 2. Materials and Methods

### 2.1. Production of mRNA and Formulation in Lipid Nanoparticles

The production process of mRNA vaccines involves synthesizing the DNA sequence encoding the HSV-2 viral protein UL41 (supplementary files, SEQ ID NO. 1), cloning it into a vector, and preparing a linearized DNA template. This template is utilized for in vitro transcription to generate mRNA. The DNA template is subsequently removed by DNase I treatment, followed by purification through LiCl precipitation, ethanol washing, and centrifugation. The UL41 mRNA is capped at the 5′ terminus, polyadenylated at the 3′ terminus, and further purified before being dissolved and quantified (supplementary files, SEQ ID NO. 2). Lipid nanoparticles (LNPs) were prepared by mixing an organic phase with an aqueous phase. The mixture was diluted with PBS, and the ethanol was removed via ultrafiltration. The LNPs encapsulate the mRNA, resulting in the final vaccine product.

The methods used to describe the synthesis of UL41 nucleoside-modified mRNA-LNPs, and improvements in their transfection efficiency and cytotoxicity are described in the legends of Figure S1 and Table S1.

### 2.2. Study Design

**Figure 1:**
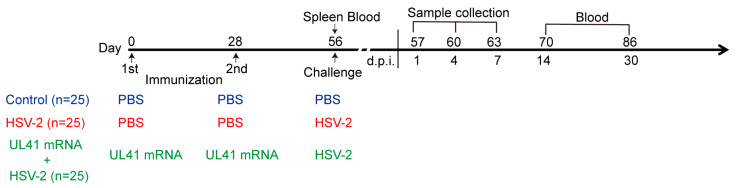
Experimental design.

Seventy-five female mice were purchased from Sibeifu Biotechnology Co., Ltd., Zhengzhou, China. These mice were aged 4 weeks. The mice were classified into three groups: the control group, the HSV-2 group, and the UL41 mRNA + HSV-2 group. Each group contained 25 mice. The mice in the UL41 mRNA + HSV-2 group were immunized twice with 10 μg of the HSV-2 UL41 mRNA vaccine at day 0 and 28 in the gastrocnemius muscle, and the mice in the HSV-2 group and control group were immunized twice with equal volumes of PBS at the indicated timepoints (0 and 28 days). Serum and splenic cells were obtained 28 days after the last immunization. The mice in the HSV-2 group and the UL41 mRNA + HSV-2 group were then challenged with 5 × 10^5^ PFU of the HSV-2 HG52 strain intravaginally, and the mice in the control group were challenged with an equal volume of PBS intravaginally (Figure 1).

The animals were weighed continuously during immunization and challenge, and the survival rate was recorded meanwhile. Spleen tissues, vaginal tissues, and sacral dorsal root ganglia (DRG) were obtained on days 1, 4, and 7 post infection, to facilitate the observation of the viral load and inflammatory profiles. The vaginas and spleens were collected and stained with hematoxylin-eosin (HE) to observe pathological changes. RT–PCR was used to examine the levels of the inflammatory cytokines IL-6, TNF-α, and IFN-γ in the vaginal tissues. Splenic cells were collected on days 1 and 7 post infection to induce their differentiation into dendritic cells (DCs) for transcriptome sequencing, and the effects of the UL41 mRNA vaccine on mouse DCs were observed. Serum was obtained on 0, 1, 7, 14, and 30 days post HSV-2 infection; the serum was then stored at −80 °C (Figure 1). At the end of the experiment, the mice were placed in the container and subjected to slow-fill carbon dioxide asphyxiation.

### 2.3. Immunology Assays

#### 2.3.1. Splenic Cell Isolation

The mice were subjected to two immunizations with either PBS or 10 µg of the HSV-2 UL41 mRNA vaccine. Five immunized mice from each group were sacrificed 28 days after the second immunization. Splenic cells were isolated from mouse spleens via an isolation kit (Solarbio Co., Ltd., Beijing, China) according to the manufacturer’s protocol.

#### 2.3.2. Cellular Immune Response via Flow Cytometry

Splenic cells were isolated from the spleens of the mice and prepared as single-cell suspensions for flow cytometric analysis. The antibodies used for labeling the cells were fluorescein isothiocyanate (FITC)-conjugated anti-mouse CD3 (Elabsceience Co., Ltd., Wuhan, China), allophycocyanin (APC) conjugated to CD4 (Elabsceience Co., Ltd., Wuhan, China), phycoerythrin (PE)-conjugated anti-CD8 (Elabsceience Co., Ltd., Wuhan, China), peridinin-chlorophyll-protein-cyanin (PerCP-Cy5.5)-conjugated anti-mouse IFN-γ (BioLegend Co., Ltd., Beijing, China), and PerCP-Cy5.5-conjugated anti-mouse IL-4 (BioLegend Co., Ltd., Beijing, China) antibodies. The cells were analyzed on a BD FACSCalibur system. Gates were set to discriminate singlet cells and then lymphocytes based on forward scatter and side scatter properties. We collected 10,000 cell events in the lymphocyte gate. After data collection, flow samples were analyzed via FlowJo version 10.8. The cells were gated on CD4^+^ or CD8^+^ T cells that produced single cytokines. Boolean combination gates were used to compare IL-4 and IFN-γ production.

### 2.4. qPCR Assay of HSV-2 DNA Present in the Vagina, Spleen and DRG

The viral loads in the tissues of three mice from each group were determined by qPCR with absolute quantification. The primers for the reaction bound within a region of the glycoprotein gene in the HSV-2 genome were as follows: forward, CGCTCTCGTAAATGCTTCCCT; reverse, TCTACCCACAACAGACCCACG; and probe, 6-FAM-CGCGGAGACATTCGAGTACCAGATCG-BHQ1. In addition, a standard curve was produced from standard DNA samples (the pUC57 plasmid was ligated to the IgG gene fragment). Viral genomic DNA was extracted from mouse tissue via a Tissue DNA Kit (Omega Biotek, Suzhou, China). The reactions were performed via the use of Premix Ex Taq on a real-time fluorescence quantitative PCR instrument (Bio-Rad, CFX96, Hercules, CA, USA).

### 2.5. Quantitative Real-Time Polymerase Chain Reaction (RT–PCR)

The mRNA levels of IL-6, TNF-α, and IFN-γ were quantified via RT–PCR. The primers for IL-6, TNF-α, and IFN-γ are listed in Table S2. Vaginas were collected from three mice per group, and total RNA was extracted using TRIzol reagent (Thermo Fisher Scientific, Waltham, MA, USA) and reverse transcribed to cDNA via a reverse transcriptase reagent kit (YEASEN Biotechnology, Shanghai, China) according to the manufacturer’s instructions. The reaction mixture for RT–PCR contained 3 μL of cDNA, 0.4 μL of each primer, 10 μL of SYBR Green mix, and 6.2 μL of water. cDNA synthesis and predenaturation were carried out at 95 °C for 2 min. Forty cycles of 95 °C for 10 s, 60 °C for 30 s, and 60 °C for 30 s were applied. The relative changes in the mRNA levels were evaluated via the 2^−ΔΔCT^ method.

### 2.6. Hematoxylin-Eosin (HE) Staining

Vaginal and spleen tissues were collected from three mice per group at 1, 4, and 7 days post challenge for pathological analysis as previously described. HE staining was performed to assess inflammation via an HE staining kit (Solarbio, Beijing, China) to assess inflammation. The tissues were fixed in 4% polymethyl aldehyde for 24~48 h, dehydrated through an ethanol gradient, embedded in paraffin, and dewaxed with xylene. The sections were incubated at 62 °C for 24 h and then rehydrated through a graded alcohol series. Hematoxylin was applied for nuclear staining, and the sections were counterstained with 5% eosin. The sections were dehydrated in 80~100% alcohol, cleared in xylene, and sealed with neutral gum.

### 2.7. Serum IgG ELISA

The mice were bled at 0, 1, 7, 14, and 30 days post HSV-2 infection. We used a Mouse Immunoglobulin G (IgG) ELISA Kit (Elabsceience Co., Ltd., Wuhan, China) to measure the level of IgG in the serum of the mice. In each group, the serum IgG level was measured in three mice. Endpoint titers in immunized mice were calculated as the dilution that had at least a 2-fold higher OD value than that of PBS control sera at the same dilution.

### 2.8. Transcriptome Profile of DCs Identified via RNA Sequencing

Splenic cells were collected from three mice per group at each time point (days 1 and 7 post challenge), with two time points and three experimental groups, for a total of eighteen mice. The splenic cells were collected by adding the cytokines GM-CSF (20 ng/mL), IL-4 (20 ng/mL), and TNF-α (20 ng/mL) to induce their differentiation into DCs for 48 h. The dendritic cells in spleen tissues may be derived from an intrasplenic precursor, a pre-cDC, which might be replenished from earlier precursor cells that are generated in the bone marrow, followed by occasional seeding of the spleen from the bloodstream, as previous research has indicated [28]. The medium was changed twice a week. After induction for 14 days, the DCs were collected for transcriptome sequencing.

### 2.9. RNA Extraction, cDNA Library Preparation, and Sequencing

Transcriptome sequencing was performed according to the protocol previously described by LC-Bio Technologies Co., Ltd. (Hangzhou, China). The DCs from differentiated splenic cells were collected (three mice per group), and total RNA was isolated according to the previously described methods. The amount and purity of the RNA were quantified and checked using a NanoDrop ND-1000 (NanoDrop, Wilmington, DE, USA). RNA integrity was assessed using a Bioanalyzer 2100 (Agilent, Santa Clara, CA, USA), and a RIN > 7.0 for each sample was confirmed by electrophoresis with a denaturing agarose gel. Poly(A) RNA was purified from 1 g of total RNA via two rounds of purification, and then fragmented into small pieces at 94 °C for 5~7 min. Taking these short fragments as templates, they were reverse-transcribed to create first-strand cDNA via SuperScript™ II Reverse Transcriptase (Invitrogen, Carlsbad, CA, USA) and random primers, followed by synthesizing second-strand DNAs via U-labeled with *E. coli* DNA polymerase I (NEB, Boston, MA, USA), RNase H (NEB, Boston, MA, USA) and dUTP Solution (Thermo Fisher, Waltham, MA, USA). The synthesized cDNA was subjected to A-base addition in the blunt ends of each strand and the ligation of indexed adapters; each adapter contains a T-base overhang for ligating the adapter to the A-tailed fragmented cDNA. Both ends of the cDNA fragments were added sequencing adapters, and size selection was performed with AMPureXP beads. After using xheat-labile UDG enzyme (NEB, Boston, MA, USA) to treat the U-labeled second-strand DNAs, the ligated products were amplified via PCR under the following conditions: initial denaturation at 95 °C for 3 min; 8 cycles of denaturation at 98 °C for 15 sec, annealing at 60 °C for 15 s, and extension at 72 °C for 30 s; and a final extension at 72 °C for 5 min. The average insert size for the final cDNA library was 250–350 bp. Finally, 2 × 150 bp paired-end sequencing (PE150) was carried out on an Illumina NovaSeq™ 6000 (LC-Bio Technology Co., Ltd., Hangzhou, China) according to the vendor’s recommended protocol.

### 2.10. Bioinformatics Analysis of RNA-Seq Data

After sequencing, the raw data were removed from the reads that contained adaptor contaminants, low-quality bases, and undetermined bases with the default parameters and quality controlled by fastp software (https://github.com/OpenGene/fastp, version number: 0.10.1, accessed on 15 January 2025). We used HISAT2 (DaehwanKimLab.github.io/hisat2, version number: 2.2.1, accessed on 15 January 2025) to align the reads to the Mus_musculus.GRCm38 reference genome. The mapped reads from each sample were assembled via StringTie (https://ccb.jhu.edu/software/stringtie, version number: 2.1.6, accessed on 15 January 2025) with default parameters, then merged to reconstruct a comprehensive transcriptome via gffcompare (https://github.com/gpertea/gffcompare/, version number: 0.12.6, accessed on 15 January 2025). After the final transcriptome data were generated, StringTie was used to estimate the expression levels of all the transcripts, and the expression of each transcript was normalized to the number of Fragments Per Kilobase of transcript per million mapped reads (FPKM) by using StringTie. The differentially expressed genes (DEGs) were selected via the criterion of an absolute value of the log_2_-transformed fold change (log_2_FC) ≥ 1 (up or down), and a parametric F test comparing nested linear models (*p* value < 0.05). DEGs were evaluated via the R package edgeR (https://bioconductor.org/packages/release/bioc/html/edgeR.html, version number: 3.22.5, accessed on 15 January 2025).

### 2.11. Statistical Analysis

The data were analyzed with GraphPad Software Prism 9.0. Comparisons were made using one-way analysis of variance (ANOVA) with Tukey’s tests. *p* < 0.05 was considered to indicate statistical significance.

## 3. Results

### 3.1. The HSV-2 UL41 mRNA Vaccine Is Nontoxic to Cells and Healthy Mice

We targeted the HSV-2 immune evasion protein UL41 and constructed an LNP-UL41 mRNA complex to generate an HSV-2 UL41 mRNA vaccine (Figure S1, Table S1). To observe the entry of LNP-UL41 mRNA into cells under a microscope, we used Luc-mRNA and UL41 mRNAs that were wrapped in LNPs at the same time. After the 293T cells were transfected with 10 µg of LNP-(UL41+Luc) for 24 h, the fluorescence intensity of the 293T cells was observed with an in vivo imager. The results revealed that the LNP-(UL41+Luc) group had stronger fluorescence, whereas the normal group had no fluorescence, indicating that LNP-(UL41+Luc) successfully entered the cells and that its transfection efficiency was high (Figure S1c). A CCK8 kit was used to detect the cytotoxicity of LNP-(UL41+Luc). Compared with the control, the addition of LNP-(UL41+Luc) had no significant effect on cell viability (Figure S1d).

**Figure 2:**
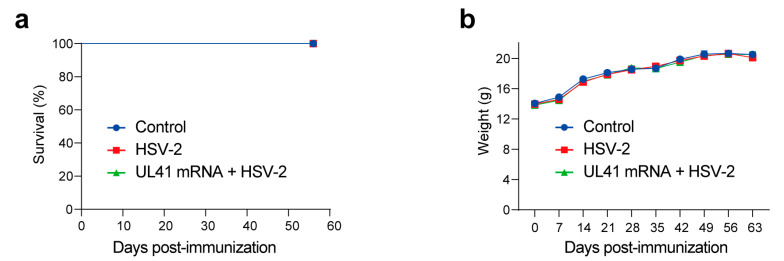
The survival rate and weights of mice in the control, HSV-2, and UL41 mRNA + HSV-2 groups during immunization. (**a**) Survival curves of the control and (**b**) weight curves of each group. *n* = 10.

We subsequently immunized the mice according to the experimental design shown in Figure 1. During this period, the survival rate and body weight of the mice were continuously observed. The results revealed that the body weights of the mice in the HSV-2 UL41 mRNA vaccine-immunized group were not affected and that no individuals died (Figure 2a,b). Therefore, in vitro and in vivo experiments have shown that the HSV-2 UL41 mRNA vaccine has a favorable safety profile.

### 3.2. The HSV-2 UL41 mRNA Vaccine Can Effectively Activate Cellular Immunity In Vivo

**Figure 3:**
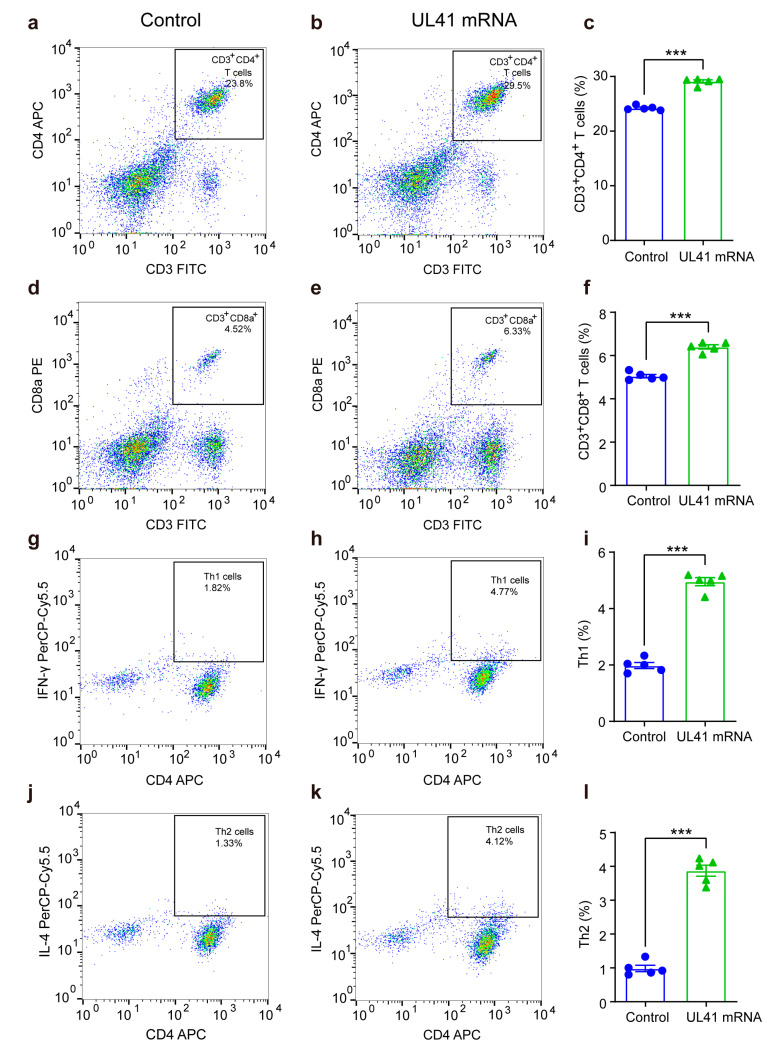
The percentages of CD3^+^CD4^+^ T cells, CD3^+^CD8^+^ T cells, CD4^+^ Th1 cells, and CD4^+^ Th2 cells in the spleen tissue of control and UL41 mRNA-immunized mice. (**a**–**c**) Flow cytometry results of CD4^+^ T cells in the control and UL41 mRNA groups. (**d**–**f**) Flow cytometry results of CD8^+^ T cells in the control and UL41 mRNA + HSV-2 groups. (**g**–**i**) Flow cytometry results of Th1 cells in the control and UL41 mRNA groups. (**j**–**l**) Flow cytometry results of Th2 cells in the control and UL41 mRNA groups. (*n* = 5). Left panel: representative images of the flow cytometry results; right panel: summarized results of flow cytometry. Two-sample t tests were used to compare the two groups, the error bars represent the SEMs, and *p* < 0.05 was considered statistically significant. *** *p* < 0.001.

The immunogenicity of an mRNA vaccine is the key to whether it can effectively activate the immune system. To evaluate the immunogenicity of the HSV-2 UL41 mRNA vaccine, we collected single cells from the spleen tissues in control and UL41 mRNA vaccine-immunized mice on day 56, four weeks after the second immunization, and observed the activation of T cells in the spleens of the mice. A gating strategy was adopted to analyze T cells as shown in Figure S2. Compared with mice in the control group, the percentages of CD4^+^ and CD8^+^ T cells (Figure 3a–f), as well as IFN-γ-secreting predominantly Th1 cells and IL-4-secreting Th2 cells, were significantly increased in the UL41 mRNA vaccine-immunized group (*p* < 0.001) (Figure 3g–l). The results indicated that the mRNA vaccine significantly increased the number of CD8^+^ T cells, CD4^+^ Th1 cells, and Th2 cells, suggesting that cellular immunity was activated in mice after immunization.

### 3.3. Vaccination Reduces the Viral Load and Inflammatory Response in Mice Subjected to HSV-2 Challenge and Protects Them from Pathogens

**Figure 4:**
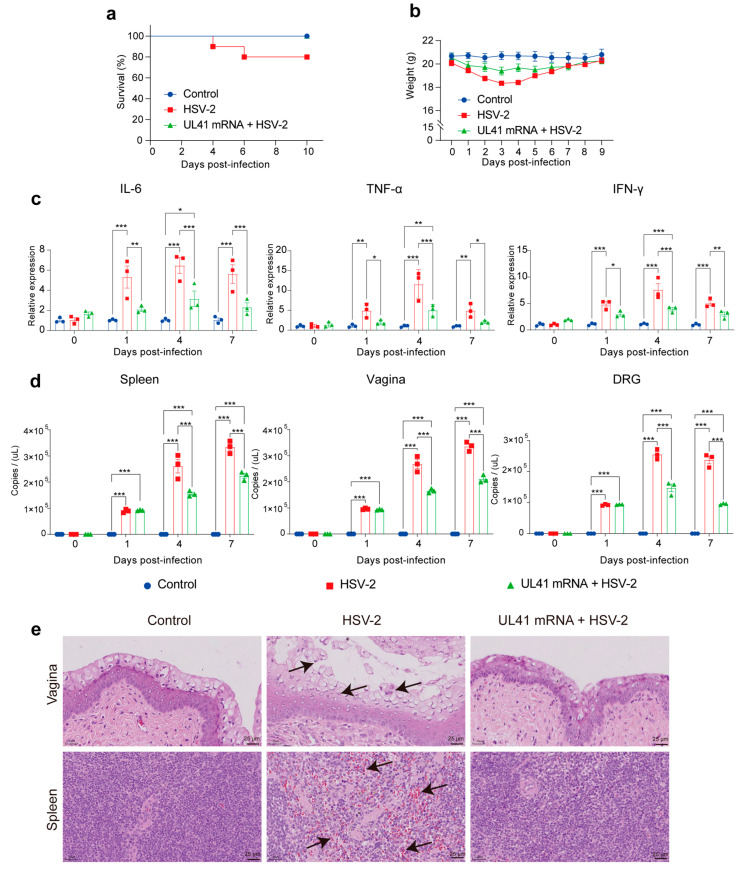
Comparison of the protective effects, inflammatory responses, and histology of mice in the control, HSV-2, and UL41 mRNA + HSV-2 groups after HSV-2 infection. (**a**) Survival curve. *p* values were calculated via the log-rank test (*n* = 10). (**b**) Weights of the mice in the control, HSV-2, and UL41 mRNA + HSV-2 groups (*n* = 10). (**c**) IL-6 (left panel), TNF-α (middle panel), and IFN-γ (right panel) levels in the vagina (*n* = 3). (**d**) HSV-2 viral loads in the spleen (left panel), vagina (middle panel), and DRG (right panel) (*n* = 3). The experiments were performed once with three mice in the PBS group, three mice in the HSV-2 group, and three mice in the HSV-2 UL41 mRNA group at days 1, 4, and 7 after the challenge. Statistical differences were determined by one-way analysis of variance (ANOVA) with Tukey’s multiple comparisons test. * *p* < 0.05, ** *p* < 0.01, *** *p* < 0.001. The error bars represent the SEMs (**c**,**d**). (**e**) UL41 mRNA improved the histopathological images by HE staining among the UL41 mRNA + HSV-2, HSV-2, and control groups. Vaginal tissues (upper panels) and spleen tissues (lower panels). The black arrows indicate the inflammtory inflitration.

Four weeks after the final immunization, the mice in the HSV-2 and UL41 mRNA + HSV-2 groups were administered a lethal dose (5 × 10^5^ PFU) of HSV-2 HG52. The mice in the control group were administered an equal volume of PBS. At 4 days post infection (d.p.i.), the survival rate of the HSV-2 group started to decrease and reached 80% at 6 d.p.i. (Figure 4a). As shown in Figure 4b, the body weights of the UL41 + HSV-2 mRNA vaccine groups decreased, and the weight of the HSV-2 group was significantly lower than that of the control group from the second to the fifth day after administration; moreover, the weights of the HSV-2 group were significantly lower than those of the UL41 mRNA + HSV-2 vaccine groups from the third and fourth days. The weight of the UL41 + HSV-2 mRNA vaccine group started to recover from the sixth day, and the weight of the UL41 + HSV-2 mRNA vaccine group decreased, but it was not significantly different from that of the normal group. On the sixth day, the weight of the UL41 + HSV-2 mRNA vaccine group decreased but was not significantly different from that of the normal group (*p* > 0.05) (Table 1). The weight of the UL41 mRNA vaccine group started to recover on the fourth day after the attack, and the immunized group did not experience any deaths, with a survival rate of 100%.

Vaginal tissues were collected to detect inflammatory cytokine levels at 1, 4, and 7 d.p.i. The forward and reverse primers used are shown Table S2. The levels of IL-6, TNF-α, and IFN-γ in the HSV-2 group were significantly greater than those in the control group (*p* < 0.01), and the levels of IL-6, TNF-α, and IFN-γ in the UL41 mRNA + HSV-2 group were significantly lower than those in the HSV-2 group at 1, 4, and 7 d.p.i. (*p* < 0.01). The levels of these three inflammatory cytokines in the UL41 mRNA + HSV-2 group were significantly greater on the fourth day than those in the control group and returned to normal levels on the seventh day (Figure 4c). Spleen, vaginal, and DRG tissues were collected to detect the viral load at 1, 4, and 7 d.p.i. The results revealed that the viral load in each tissue of the HSV-2 group was significantly greater than that in the control group (*p* < 0.001). The viral load in the UL41 vaccine mRNA-immunized group was significantly greater than that in the control group at 1, 4, and 7 d.p.i., but significantly lower than that in the HSV-2 group at 4 and 7 d.p.i. (*p* < 0.001) (Figure 4d). These results show that immunization with the UL41 mRNA vaccine can inhibit virus replication in various tissues after HSV-2 infection. Compared with those in the control group, the epithelial cells in the vaginal tissues of the HSV-2 group shed, inflammatory cells infiltrated, and the spleen tissues exhibited bleeding and inflammatory cell infiltration. Compared with the HSV-2 group, the UL41 mRNA + HSV-2 group showed milder symptoms in the vaginal and spleen tissues (Figure 4e). The results showed that the HSV-2 UL41 mRNA vaccine activated cellular immunity and alleviated the inflammatory response in mice following HSV-2 infection, which was related to the modulation of the host immune response by UL41, including both innate and adaptive immunity.

### 3.4. UL41 mRNA Vaccine Protection Is Independent of Anti-HSV-2 IgG Antibodies

**Figure 5:**
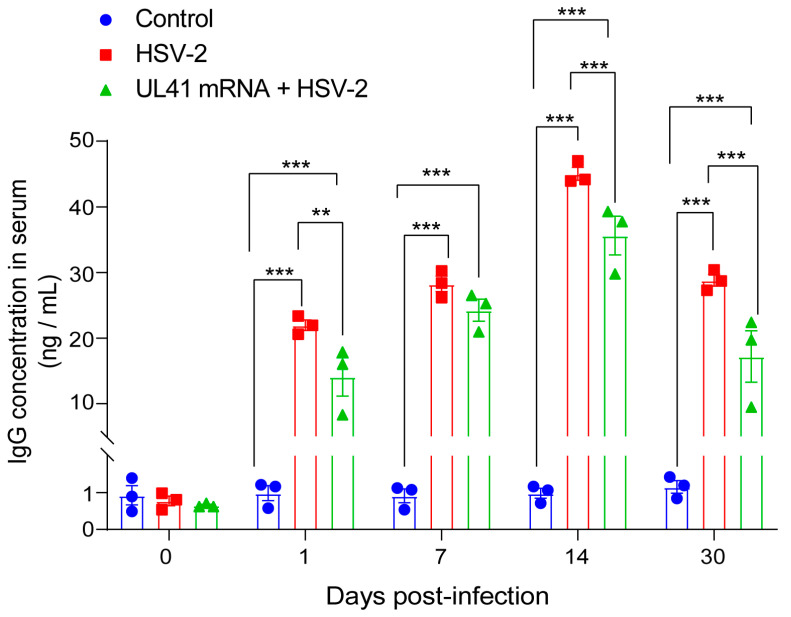
The IgG levels in the serum of mice in the control, HSV-2, and UL41 mRNA + HSV-2 group after HSV-2 infection. The serum IgG levels of the mice were measured via enzyme-linked immunosorbent assay (ELISA) (*n* = 3 ). The experiments were performed once at 0, 1, 7, 14, and 30 days after the challenge. Statistical differences were determined by one-way analysis of variance (ANOVA) with Tukey’s multiple comparisons test. ** *p* < 0.01, *** *p* < 0.001. The error bars represent the SEM.

To test whether the UL41 mRNA vaccine induces humoral immunity in mice. We collected serum from each group of mice at 0, 1, 7, 14, and 30 days post HSV-2 infection for IgG detection. The results revealed that the UL41 mRNA vaccine could decrease IgG antibody production in vivo after HSV-2 infection, and the level of IgG production was significantly greater in the HSV-2 group than that in the UL41 mRNA + HSV-2 group one day, two weeks, and one month after administration (*p* < 0.01) (Figure 5). Although the IgG levels were greater in the HSV-2 group than in the UL41 mRNA + HSV-2 group, as determined via body weights and survival curves, the high levels of IgG antibodies did not exert the appropriate protective effects, and approximately 20% of the mice died. In contrast, the UL41 mRNA vaccine-immunized group with low levels of IgG had a 100% survival rate. These experimental results suggest that the protective effect of the UL41 mRNA vaccine does not depend on the humoral immunity activated by the UL41 mRNA vaccine, but rather on the cellular immunity activated by the UL41 mRNA vaccine.

### 3.5. RNA Sequencing Analysis Revealed a Differential Transcriptome Profile in DCs from the Mouse Spleen

To study the possible mechanism by which DCs affect HSV2 UL41 mRNA vaccine immunity, we injected mice with the mRNA vaccine and then used the HSV-2 wild-type virus for vaginal challenge after immunization. One day and seven days after the challenge, the spleens were isolated from the mice, and the splenic cells were extracted, mouse splenic cells were induced to differentiate into DCs (Figure S3). The DCs were subjected to transcriptome sequencing. A series of data processing and screening methods, such as total RNA extraction and quality inspection, library construction and evaluation, sample sequencing and screening, reference sequence comparison and analysis, gene expression level analysis, and gene expression difference analysis, were used to identify DEGs and significant signaling pathways related to HSV2 mRNA vaccine immunity.

**Figure 6:**
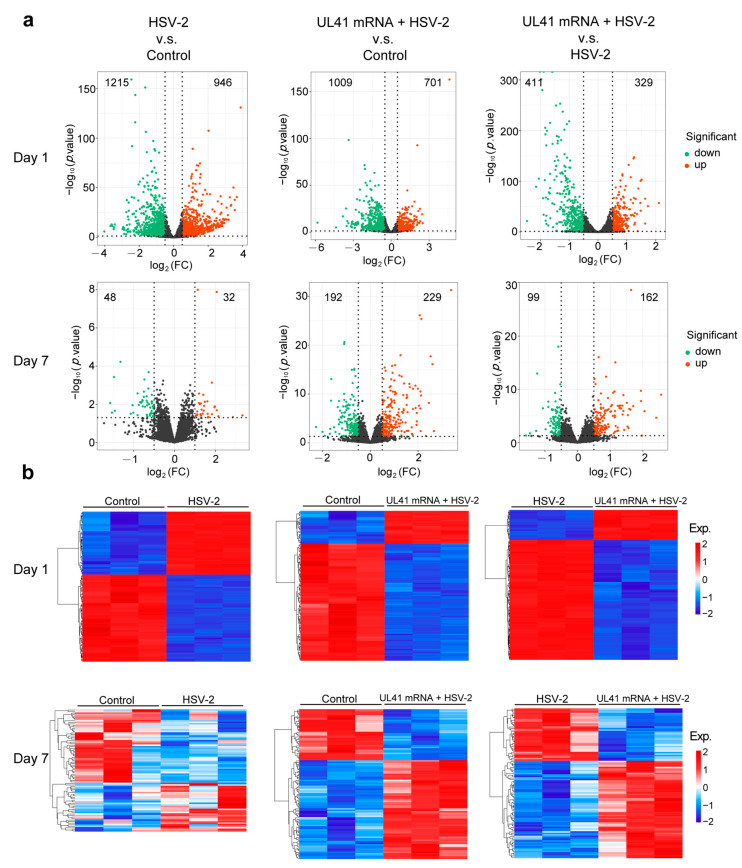
The significantly differentially expressed genes of HSV-2 vs. control, UL41 mRNA + HSV-2 vs. control, and UL41 mRNA +HSV-2 vs. HSV-2. (**a**) Volcano maps of different genes at 1 and 7 d.p.i. according to pairwise comparisons among the control, HSV-2, and UL41 mRNA + HSV-2 groups. (**b**) Heatmaps of DEGs at 1 and 7 d.p.i. according to pairwise comparisons among the control, HSV-2, and UL41 mRNA + HSV-2 groups. Red represents upregulated proteins, and green or blue represents downregulated genes.

To visually observe the DEGs and eliminate biological variation, this study evaluated the HSV-2 group and control group, the UL41 mRNA + HSV-2 group, and the HSV-2 group from two aspects: differences in the fold change and significance level. The DEGs were screened, and volcano plots were drawn, as shown in Figure 6a. The analysis threshold for this experiment was set to |log_2_FoldChange| ≥ 1 and *p* < 0.05. The number of DEGs on day 1 after the challenge was significantly greater than that after 7 days post administration.

According to the expression level of DEGs in each sample, after taking the logarithm with base 2, the DEGs were clustered via the hierarchical cluster method. The final clustering result of the DEGs is shown in Figure 6b, Table S3. Genes or samples with similar expression patterns in the heatmap were clustered together. The color in each square does not reflect the gene expression value but rather the value obtained after normalization of the expression data. The abscissa of the heatmap is the sample, and the ordinate is the filtered DEGs (the default top 100 with the smallest *p* value is used as an example of the heatmap). The results revealed that the DEGs in the HSV-2 group and UL41 mRNA + HSV-2 group were significantly increased compared with those in the control group.

**Figure 7:**
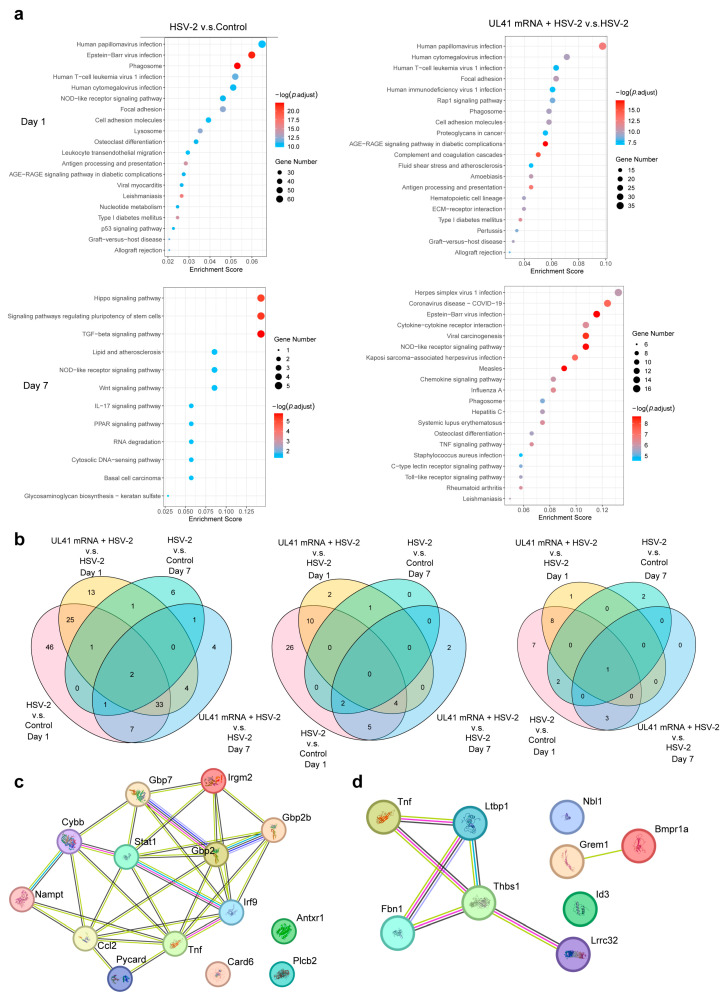
Enriched functional terms and the interactions of crossover DEGs within the selected pathways in DCs during HSV-2 infection. (**a**) Target KEGG enrichment analysis plot. (**b**) Venn diagrams and crossover plots of signaling pathways associated with HSV-2 and UL41 mRNA targets. (**c**) PPI network plots of crossover targets in the STRING database of the NLR signaling pathway. (**d**) PPI network plots of crossover targets in the STRING database of the TGF-β signaling pathway.

The DEGs were subjected to Kyoto Encyclopedia of Genes and Genomes (KEGG) enrichment analysis via the KEGG Orthology-Based Annotation System (KOBAS), and the top 20 pathways with the greatest significance are displayed in Figure 7a. On the first day after immunization, the HSV-2 group vs. the control group was enriched in a total of 328 signaling pathways, of which 115 were significant (*p* < 0.05) pathways, including the phagosome pathway, antigen processing and presentation pathway, the nod-like receptor signaling pathway, the PI3K–Akt signaling pathway, human cytomegalovirus infection, leukocyte transendothelial cell migration, natural killer (NK) cell-mediated cytotoxicity, Th1 and Th2 cell differentiation, etc. A total of 292 signaling pathways were enriched from the UL41 mRNA + HSV-2 group vs. HSV-2 group, of which 79 were greatest significance (*p* < 0.05) pathways, including the complement and coagulation cascade, antigen processing and extraction, cell adhesion molecules, the PI3K-Akt signaling pathway, the ECM–receptor interaction, the JAK-STAT signaling pathway, the interaction between viral proteins and cytokines, and cytokine and cytokine receptors, and natural killer cell-mediated cytotoxicity.

On the seventh day after immunization, a total of 71 signaling pathways, including the TGF-β signaling pathway, the signaling pathway that regulates stem cell pluripotency, the Wnt signaling pathway, the PPAR signaling pathway, the Nod-like receptor signaling pathway, the IL-17 signaling pathway, etc., were enriched in the DEGs from the HSV-2 group vs. the control group. A total of 200 signaling pathways from the UL41 mRNA + HSV-2 group vs. the HSV-2 group were significantly enriched, and 52 pathways (*p* < 0.05), including cytokine–cytokine receptor interaction, Th17 cell differentiation, the TNF signaling pathway, the Toll-like receptor signaling pathway, the JAK-STAT signaling pathway, the IL-17 signaling pathway, and the interaction of viral proteins with cytokines and cytokine receptors, were enriched.

### 3.6. Screening of DC Characteristic Genes After UL41 mRNA Vaccine Immunization

To screen the characterized genes according to the intervention conditions of the model, we intersected the pathways with significant enrichment (*p* < 0.05) of the four groups of DEGs via KEGG and identified two common pathways, namely, the nod-like receptor signaling pathway and the TGF-β pathway.

To check the DEGs that were coenriched in the same pathway among the groups, we performed a Venn analysis on the DEGs that were enriched in the same pathway in each group (Figure 7b). The results revealed that there were no DEGs enriched in the nucleotide-binding oligomerization domain (NOD)-like receptor (NLR) signaling pathway among the four groups. A total of 14 DEGs were enriched in the NLR signaling pathway in the two groups immunized for 1 day, and a total of 9 DEGs were enriched in the TGF-β signaling pathway, whereas only 14 DEGs were enriched in the two groups that were immunized for 7 days. Two DEGs were enriched in the NLR signaling pathway at the same time; a common *Id3* gene among the four groups was also enriched in the TGF-β signaling pathway. We also examined the expression trends of DEGs that were collectively enriched in the same pathway. Except for the *TNF* gene in the one-day immunization group, all the other genes tended to be downregulated after immunization in the HSV-2 group or upregulated after immunization in the HSV-2 group. However, after immunization for 7 days, none of the shared DEGs in the day group showed such a trend.

To view the relationships of the genes associated with the shared pathways at the protein level, we used the STRING database to conduct interaction network analysis. The results revealed that most of the 14 shared DEGs in the NLR signaling pathway had interactive relationships (Figure 7c). Among the nine genes involved in the TGF-β signaling pathway, five had interactive relationships (Figure 7d). We ranked the DEGs according to the connectivity between DEGs in the interaction network. The DEGs with the highest connectivity in the NLR signaling pathway were Stat1 and TNF (Table S4), and the genes with the highest connectivity in the TGF-β signaling pathway were Thbs1 (Table S5).

## 4. Discussion

In this study, we investigated the humoral and cellular responses of a mRNA vaccine based on HSV-2 UL41. This UL41 mRNA vaccine can induce greater cellular immune responses than the wild-type HSV-2 infection without vaccine immunization can. To date, this is the first report of a direct mRNA vaccine using the HSV-2 UL41 immune evasion protein. This work provides a strong foundation for optimizing better vaccines against HSV-2. To gain a further understanding of the HSV-2 UL41 mRNA vaccine, we used a viral challenge study in an animal model. Although the HSV-2 UL41 mRNA vaccine immunization group stimulated lower levels of IgG antibodies than the wild-type HSV-2 group did in the animal experiments, we believe that challenging studies can reveal more important details. We consider the HSV-2 UL41 mRNA vaccine to be a promising candidate vaccine for the prevention of HSV-2 genital herpes in clinical trials.

In recent years, mRNAs have increasingly been utilized in clinical studies across various fields, including immunology, oncology, vaccines, and innate metabolic diseases [29,30,31,32,33]. mRNA vaccines introduce mRNAs that encode disease-specific antigens into the body, leveraging the protein synthesis mechanisms of host cells to produce these antigens [34,35,36,37]. This process triggers immune responses, thereby facilitating disease prevention [31,37,38]. The investigation of HSV-2 mRNA vaccines has yielded several important findings [39,40]. Data from animal studies conducted by Friedman et al. [41,42] reported that vaccination with an HSV-2 trivalent mRNA vaccine, which included the HSV-2 gC2, gD2, and gE2 proteins, stimulated the immune system to prevent diseases associated with HSV-2 infection. However, a significant limitation of this study is the small sample size of the animal subjects, as the animals underwent vaginal biopsies following each medroxyprogesterone challenge, which may have increased their susceptibility to infection. Weissman et al. [43] employed mRNA technology to express three viral proteins in vivo, and the experimental data indicated that in both mice and guinea pigs, the protective efficacy of the HSV-2 trivalent mRNA-LNP vaccine surpassed that of the trivalent protein vaccine (which utilized CpG and alum as adjuvants). The two vaccines exhibited different levels of effectiveness in preventing subclinical symptoms associated with viral infection; however, neither was able to eliminate the occurrence of these symptoms. As research advances in understanding the pathogenesis of HSV-2, including its gene and protein functions and the host immune response, the development of safer and more effective HSV-2 mRNA vaccines to control the dissemination of the virus appears promising.

This study has several limitations. First, we included nonviral mRNA-LNPs as a negative control only in cell-based experiments, not in animal studies. This restricts the comparability of the results and hinders a full assessment of the role of the LNP-mRNA platform. Second, although we demonstrated the potential role of the LNP-UL41-mRNA vaccine in immune protection, we did not comprehensively assess the immunogenicity of UL41 or the specific immune responses against UL41. Owing to the experimental constraints, we were unable to measure antibody responses to UL41, HSV-2 neutralization, or antibody-dependent cellular cytotoxicity (ADCC) effects. Third, we used a relatively low amount of viral inoculum to evaluate the vaccine under milder infection conditions. While this minimized severe pathological responses, it may have limited the ability to detect the protective effects of the vaccine under increased viral challenge. Finally, despite vaccination with LNP-UL41-mRNA, HSV-2 DNA was still detected in the ganglia of vaccinated mice, suggesting that the vaccine did not fully prevent HSV-2 infection or latency establishment. Despite these limitations, this study provides valuable preliminary data on the potential roles of the LNP-UL41-mRNA vaccine in activating adaptive immunity and conferring partial resistance to HSV-2 infection. These findings contribute to the ongoing development of HSV-2 vaccines and offer directions for future research to increase vaccine efficacy.

Based on the results of these experiments and other scholars’ explorations of HSV mRNA vaccines, we can be sure that an mRNA vaccine made of either the HSV envelope glycoprotein or capsid protein is effective in resisting diseases associated with HSV-2 infection. However, the specific mechanism of mRNA vaccine action requires further exploration.

In summary, the HSV-2 mRNA vaccine, which specifically targets the UL41 protein, has promising potential roles in activating adaptive immune responses and providing partial protection against HSV-2 infection. While the findings from this study underscore the vaccine’s ability to stimulate immune activity, there are limitations regarding its capacity to prevent viral infection completely and control latency. Future research should focus on optimizing the vaccine platform, including the use of higher viral doses in animal models, the incorporation of additional controls, and the evaluation of more detailed immune responses, such as neutralization and antibody-dependent cellular cytotoxicity (ADCC). Despite these challenges, this study offers valuable insights into the development of mRNA vaccines for HSV-2, paving the way for more effective strategies in controlling the virus and its associated diseases.

## Figures and Tables

**Table 1:** Body weights of the mice in different groups within 7 days post infection.

Days Post Infection	Control	HSV-2	mRNA + HSV-2
0	20.668 ± 1.375	20.08 ± 1.175	20.524 ± 1.711
1	20.72 ± 1.175	19.435 ± 0.934 **	19.865 ± 1.591
2	20.54 ± 1.36	18.753 ± 0.78 **	19.713 ± 1.344
3	20.706 ± 1.284	18.346 ± 0.682 ***	19.393 ± 1.303 ^#^
4	20.689 ± 1.268	18.406 ± 0.647 ***	19.673 ± 1.271 ^#^
5	20.66 ± 1.335	18.99 ± 0.566 *	19.49 ± 1.009
6	20.55 ± 1.279	19.34 ± 0.592	19.71 ± 0.988
7	20.52 ± 1.451	19.87 ± 0.646	19.78 ± 0.951

Note: * represents *p* < 0.05 in the comparison with the control group; ** represents *p* < 0.01 in the comparison with the control group and *** represents *p* < 0.001 in the comparison with the control group; ^#^ represents *p* < 0.05 in the comparison with the HSV-2 group (mean ± SD).

## Data Availability

The authors will provide the original data that underpins the conclusions of this article without undue retention. The raw sequence data have been submitted to the NCBI Short Read Archive (SRA), the accession number of SRA is PRJNA1186989.

## Supplementary Materials

The following supporting information can be downloaded at: https://www.mdpi.com/article/10.3390/vaccines13030271/s1, supplementary files, SEQ ID NO. 1; supplementary files, SEQ ID NO. 2; Figure S1: The construction strategy of HSV-2 UL41 mRNA vaccine and cell viability followed by transfected with the LNP-(UL41+Luc) vaccine; Figure S2: Gating strategy of CD3^+^ CD4^+^ T cells, CD3^+^ CD8^+^ T cells, CD4^+^ Th1 cells and CD4^+^ Th2 cells; Figure S3: Images of DC induced differentiation results on the first and seventh days after the HSV-2 challenge in each group; Table S1: HSV-2 UL41 mRNA product test results; Table S2: Primers used in this study; Table S3: Heatmap of differential gene expression information (Excel file). Table S4: NLR signaling pathway gene connectivity and fold differences; Table S5: TGF-β signaling pathway gene connectivity and fold differences.
