# Supplementary material for: Immunogenicity and Protective Efficacy of an mRNA Vaccine Targeting HSV-2 UL41 in Mice
Source: Vaccines (Basel). 2025 Mar 5;13(3):271. doi: 10.3390/vaccines13030271 (PMC11945300; doi:10.3390/vaccines13030271)
Supplement: Supplementary file 1 [file vaccines-13-00271-s001.zip › SEQ ID NO. 1.pdf]

The full HSV-2 *UL41* coding sequence:

ATGGGTCTGTTTGGCATGATGAAGTTTGCCCAGACTCACCATCTGGTGAAG
CGCCGGGGCCTCCGGGCCCCGGAGGGCTACTTTACCCCCATCGCCGTGGAC
CTGTGGAATGTCATGTATACCCTGGTGGTTAAATATCAGCGCCGCTACCCAA
GTTACGACCGCGAGGCAATCACGCTACACTGTCTCTGTAGTATGTTACGGG
TGTTTACCCAAAAGTCCCTGTTCCCCATCTTCGTGACCGATCGCGGGGTCG
AGTGTACCGAGCCGGTTGTGTTCGGGGCCAAGGCGATCCTGGCCCGCACG
ACGGCCCAGTGCCGCACGGACGAGGAGGCCAGTGACGTAGACGCCTCGCC
GCCGCCTTCCCCCATCACCGACTCCAGGCCCAGTTTCGCCTTTTCCAACAT
GCGCCGCCGCGGGCACGCCTTCGCCCCGGGGGACCGGGGAACGCGGGCC
GCCGGCCCAGGCCCGGCGGCCCCCTCGGGCGCGCCCTCGAAGCCGGCCCT
GCGCCTGGCTCACCTGTTCTGTATCCGCGTTCTGCGGGCGCTGGGGTACGC
CTACATCAACTCGGGTCAGCTGGAGGCCGACGACGCCTGCGCGAACCTCTA
TCATACCAACACGGTCGCGTACGTGCATACCACGGATACCGATCTCCTGCTG
ATGGGCTGCGATATCGTGTTGGACATCAGCACCGGCTACATTCCGACGATTC
ACTGCCGCGACCTGCTGCAGTACTTCAAGATGAGTTACCCGCAGTTCCTGG
CGCTGTTCGTCCGCTGCCACACAGACCTGCACCCCAATAACACCTACGCGT
CCGTCGAGGACGTGCTGCGCGAGTGTCACTGGACCGCCCCGAGCCGATCC
CAGGCCCGCCGGGCGGCCCGGCGGGAGCGCGCCAACTCGCGCTCCCTGGA
GAGCATGCCTACGCTGACCGCGGCCCCGGTCGGCCTCGAGACGCGCATCTC
GTGGACCGAAATTCTGGCCCAACAGATCGCGGGCGAGGACGACTACGAAG
AAGACCCCCCCCTCCAGCCCCCGGACGTCGCCGGTGGGCCGCGCGACGGC
GCCCGGTCGTCCTCCTCGGAGATACTCACCCCGCCCGAGCTCGTGCAGGTC
CCCAACGCGCAGCGGGTCGCGGAACACCGCGGCTATGTCGCCGGACGTCG
CCGCCACGTCATCCACGACGCCCCGGAGGCCCTGGACTGGCTGCCCGATC
CGATGACCATCGCCGAGCTGGTGGAGCACAGATACGTCAAGTACGTCATAT
CGCTTATCAGCCCCAAGGAGCGGGGACCCTGGACTCTTCTAAAAAGACTG
CCCATCTATCAGGACCTCCGCGACGAAGATTTAGCGCGCTCCATCGTGACT
CGGCATATCACCGCCCCGGACATCGCCGACCGGTTTCTGGCGCAGCTGTGG
GCCCACGCGCCCCCGCCCGCGTTTTACAAGGACGTCCTGGCTAAATTCTGG
GACGAGTAG
