# Supplementary material for: Immunogenicity and Protective Efficacy of an mRNA Vaccine Targeting HSV-2 UL41 in Mice
Source: Vaccines (Basel). 2025 Mar 5;13(3):271. doi: 10.3390/vaccines13030271 (PMC11945300; doi:10.3390/vaccines13030271)
Supplement: Supplementary file 1 [file vaccines-13-00271-s001.zip › SEQ ID NO. 2.pdf]

The sequence of HSV-2 *UL41* added by Cap and poly(A):

GAATTCGAAATTAATACGACTCACTATAGGGAAATAAGAGAGAAAAGAAGA
GTAAGAAGAAATATAAGAGCCACCATGGGTCTGTTTGGCATGATGAAGTTT
GCCCAGACTCACCATCTGGTGAAGCGCCGGGGCCTCCGGGCCCCGGAGGG
CTACTTTACCCCCATCGCCGTGGACCTGTGGAATGTCATGTATACCCTGGTG
GTTAAATATCAGCGCCGCTACCCAAGTTACGACCGCGAGGCAATCACGCTA
CACTGTCTCTGTAGTATGTTACGGGTGTTTACCCAAAAGTCCCTGTTCCCCA
TCTTCGTGACCGATCGCGGGGTCGAGTGTACCGAGCCGGTTGTGTTCGGGG
CCAAGGCGATCCTGGCCCGCACGACGGCCCAGTGCCGCACGGACGAGGA
GGCCAGTGACGTAGACGCCTCGCCGCCGCCTTCCCCCATCACCGACTCCAG
GCCCAGTTTCGCCTTTTCCAACATGCGCCGCCGCGGGCACGCCTTCGCCCC
GGGGGACCGGGGAACGCGGGCCGCCGGCCCAGGCCCGGCGGCCCCCTCG
GGCGCGCCCTCGAAGCCGGCCCTGCGCCTGGCTCACCTGTTCTGTATCCGC
GTTCTGCGGGCGCTGGGGTACGCCTACATCAACTCGGGTCAGCTGGAGGC
CGACGACGCCTGCGCGAACCTCTATCATACCAACACGGTCGCGTACGTGCA
TACCACGGATACCGATCTCCTGCTGATGGGCTGCGATATCGTGTTGGACATC
AGCACCGGCTACATTCCGACGATTCACTGCCGCGACCTGCTGCAGTACTTC
AAGATGAGTTACCCGCAGTTCCTGGCGCTGTTCGTCCGCTGCCACACAGAC
CTGCACCCCAATAACACCTACGCGTCCGTCGAGGACGTGCTGCGCGAGTGT
CACTGGACCGCCCCGAGCCGATCCCAGGCCCGCCGGGCGGCCCGGCGGGA
GCGCGCCAACTCGCGCTCCCTGGAGAGCATGCCTACGCTGACCGCGGCCC
CGGTCGGCCTCGAGACGCGCATCTCGTGGACCGAAATTCTGGCCCAACAG
ATCGCGGGCGAGGACGACTACGAAGAAGACCCCCCCCTCCAGCCCCCGGA
CGTCGCCGGTGGGCCGCGCGACGGCGCCCGGTCGTCCTCCTCGGAGATAC
TCACCCCGCCCGAGCTCGTGCAGGTCCCCAACGCGCAGCGGGTCGCGGAA
CACCGCGGCTATGTCGCCGGACGTCGCCGCCACGTCATCCACGACGCCCCG
GAGGCCCTGGACTGGCTGCCCGATCCGATGACCATCGCCGAGCTGGTGGA
GCACAGATACGTCAAGTACGTCATATCGCTTATCAGCCCCAAGGAGCGGGG
ACCCTGGACTCTTCTAAAAAGACTGCCCATCTATCAGGACCTCCGCGACGA
AGATTTAGCGCGCTCCATCGTGACTCGGCATATCACCGCCCCGGACATCGC
CGACCGGTTTCTGGCGCAGCTGTGGGCCCACGCGCCCCCGCCCGCGTTTTA
CAAGGACGTCCTGGCTAAATTCTGGGACGAGTAGAGCGGCCGCGACTCTA
GATCATAATCAGCCATACCACATTTGTAGAGGTTTTACTTGCTTTAAAAAAC

CTCCCACACCTCCCCCTGAACCTGAAACATAAAATGAATGAAAAAAAAAAA
AAAAAAAAAAAAAAAAAAAAAAAGCTT
