# Supplementary material for: Immunogenicity and Protective Efficacy of an mRNA Vaccine Targeting HSV-2 UL41 in Mice
Source: Vaccines (Basel). 2025 Mar 5;13(3):271. doi: 10.3390/vaccines13030271 (PMC11945300; doi:10.3390/vaccines13030271)
Supplement: Supplementary file 1 [file vaccines-13-00271-s001.zip › vaccines-3455486-supplementary materials.pdf]

Supplementary materials

Immunogenicity and protective efficacy of an mRNA vaccine targeting HSV-2 UL41 in mice

Mou et al. Submission, 2024

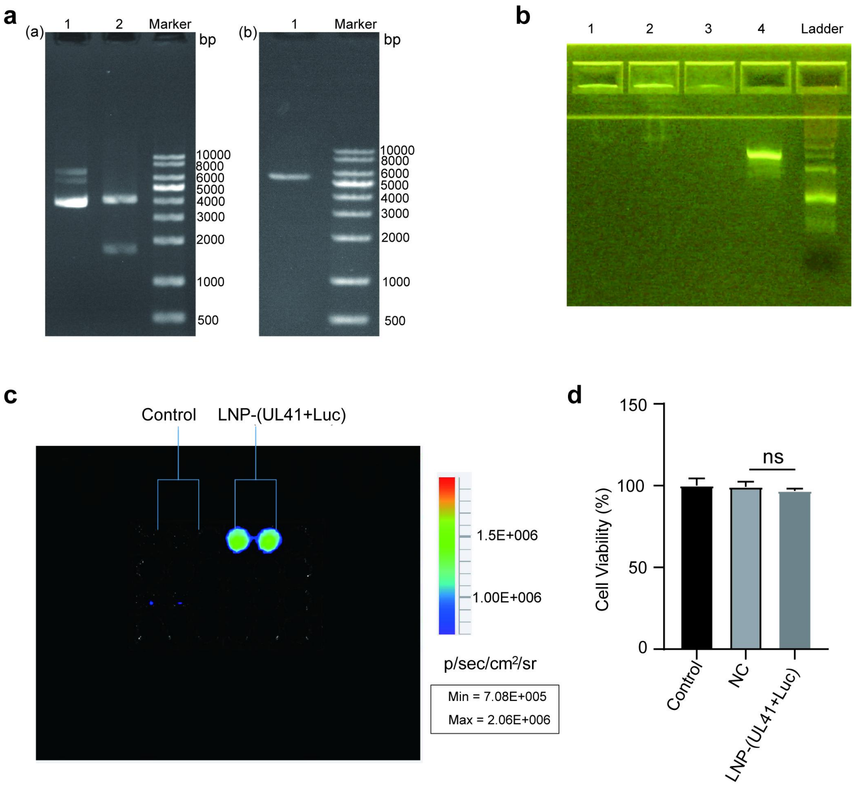

Figure S1. The construction strategy of HSV-2 UL41 mRNA vaccine and cell viability followed by transfected with the LNP-(UL41+Luc) vaccine. (**a**) Linearization results of recombinant plasmid V0301-UL41 double digestion identification and in vitro transcription template DNA. a Lane 1: V0301 plasmid; Lane 2: agarose gel electrophoresis of the recombinant V0301-UL41 plasmid for enzymatic identification. b Lane 1: Linearization results of in vitro transcribed template DNA. Marker: KB ladder 10000. (**b**) Gel retardation gel diagram. Lane 1: LNP-UL41 mRNA complex (1 μg), Lane 2: LNP-UL41 mRNA complex (2 μg), Lane 3: LNP-UL41 mRNA complex (2 μg) + RNase. Lane 4: Negative control. Lane 5: Ladder. (**c**) Fluorescence observation of HEK293T cells transfected with the LNP-(UL41+Luc) vaccine. (**d**) Toxicity of LNP-(UL41+Luc) to HEK293T cells.

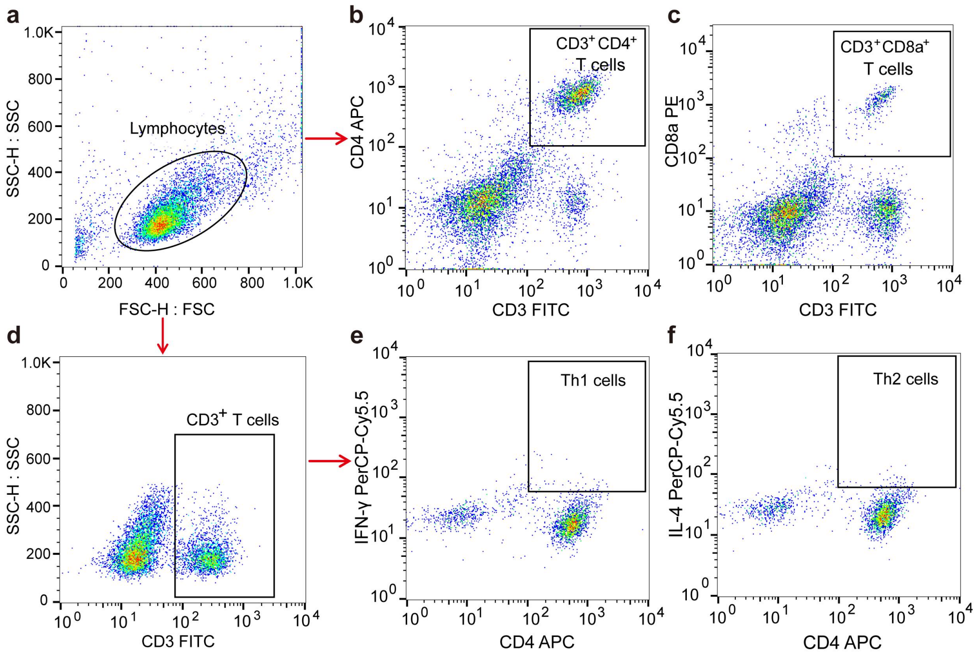

Figure S2. Gating strategy of CD3⁺ CD4⁺ T cells, CD3⁺ CD8⁺ T cells, CD4⁺ Th1 cells and CD4⁺ Th2 cells. (**a**) Gating strategy for lymphocytes. (**b**) Gating strategy used for analysis of CD4⁺ T cells. (**c**) Gating strategy used for analysis of CD8⁺ T cells. (**d**) Gating strategy used for the analysis of CD3⁺ T cells. (**e**) Gating strategy used for analysis of Th1 cells. (**f**) Gating strategy used for analysis of Th2 cells. FSC, forward scatter; SSC, side scatter.

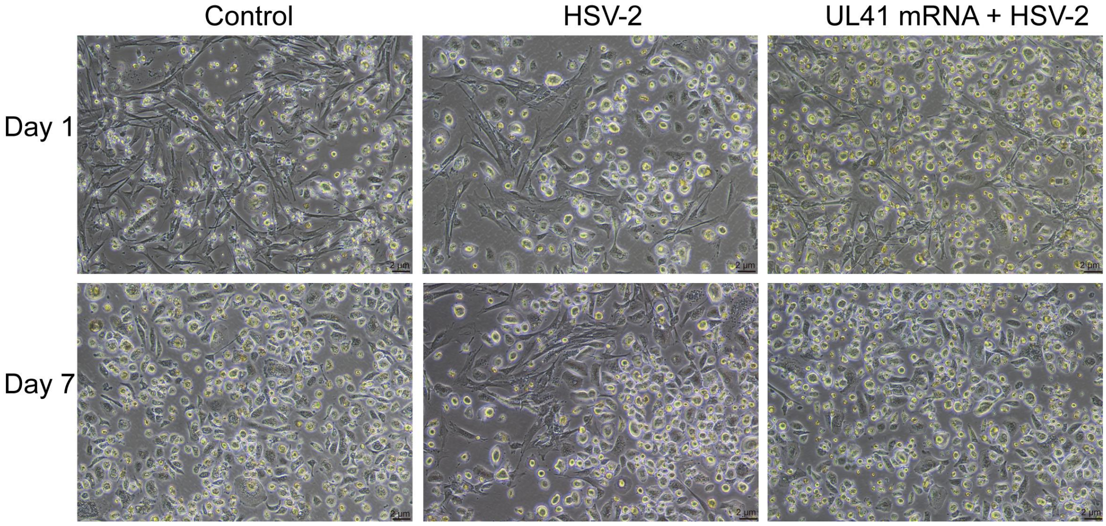

Figure S3. Images of DC induced differentiation results on the first and seventh days after the HSV-2 challenge in each group. Magnification ×100.

Table S1. HSV-2 UL41 mRNA product test results.

Item	Result	Standard	Conclusion
Appearance	Clarity and transparency	Clarity and transparency	Qualified
crude extract purity	90.10%	≥80%	Qualified
Cap rate	95.10%	≥90%	Qualified
Concentration	2 mg/ml	2 mg/ml	Qualified

Table S2. Primers used in this study.

Gene name	Forward primers (5′-3′)	Reverse primers (5′-3′)
IL-6	CCACCGGGAACGAAAGAGAA	TTCTCCTGGGGGTATTGTGGA
TNF-α	GAGGACCAAGGAGGAAAGTATG	GGTCTTGTAGATGGACTGTTTACT
IFN-γ	AAATCCTGCAGAGCCAGATTAT	GCTGTTGCTGAAGAAGGTAGTA
GAPDH	CCTTCCGTGTTCCTACCCC	GCCCAAGATGCCCTTCAGT

Table S3. Heatmap of differential gene expression information. For detailed information, please refer to Supplementary Material Table A3 (Excel file).

Table S4. NLR signaling pathway gene connectivity and fold differences.

Name	Degree	Day 1 (log_2FC)	
		HSV-2 v.s. Control	UL41 mRNA + HSV-2 v.s. HSV-2
Stat1	8	-1.752337019	0.924268303
Tnf	8	-1.124128717	-0.504678581
Ccl2	7	0.81627766	-0.812872546
Irf9	7	-1.400705092	0.508485334
Gbp2	7	-2.611991998	0.855562425
Cybb	5	-1.560194922	0.533029149
Gbp7	5	-1.303686008	0.954500261
Gbp2b	5	-2.295112927	1.000420854
Irgm2	5	-2.385159854	0.714523803
Nampt	3	-0.911621664	0.518543968
Pycard	2	-0.903774787	0.516144708

Table S5. TGF-β signaling pathway gene connectivity and fold differences.

Name	Degree	Day 1 (log_2FC)	
		HSV-2 v.s. Control	UL41 mRNA + HSV-2 v.s. HSV-2
Thbs1	4	1.178722552	-1.083240636
Ltbp1	3	1.472976991	-1.517085065
Fbn1	2	2.86701969	-1.306446579
Tnf	2	-1.124128717	-0.504678581
Bmpr1a	1	1.000197822	-0.650446423
Grem1	1	1.979919933	-0.530455913
Lrrc32	1	2.276127537	-1.502373286
